# Zhen Gan Xi Feng Decoction, a Traditional Chinese Herbal Formula, for the Treatment of Essential Hypertension: A Systematic Review of Randomized Controlled Trials

**DOI:** 10.1155/2013/982380

**Published:** 2013-03-20

**Authors:** Xingjiang Xiong, Xiaochen Yang, Bo Feng, Wei Liu, Lian Duan, Ao Gao, Haixia Li, Jizheng Ma, Xinliang Du, Nan Li, Pengqian Wang, Kelei Su, Fuyong Chu, Guohao Zhang, Xiaoke Li, Jie Wang

**Affiliations:** ^1^Department of Cardiology, Guang'anmen Hospital, China Academy of Chinese Medical Sciences, Beixiange 5, Xicheng District, Beijing 100053, China; ^2^Department of Gastroenterology, Guang'anmen Hospital, China Academy of Chinese Medical Sciences, Beijing 100053, China; ^3^Graduate School, China Academy of Chinese Medical Sciences, Beijing 100700, China; ^4^Department of Endocrinology, Traditional Chinese Medicine Hospital of Mentougou District, Beijing 102300, China; ^5^The First Clinical Medical College, Nanjing University of Chinese Medicine, Jiangsu 210029, China; ^6^Department of Cardiology, Beijing Traditional Chinese Medicine Hospital, Capital Medical University, Beijing 100010, China; ^7^Department of Cardiology, Worker's Hospital of Kweichow Moutai Co., Ltd., Guizhou 564501, China; ^8^Basic Medical College, Beijing University of Chinese Medicine, Beijing 100029, China

## Abstract

*Objectives*. To assess the clinical effectiveness and adverse effects of Zhen Gan Xi Feng Decoction (ZGXFD) for essential hypertension (EH). *Methods*. Five major electronic databases were searched up to August 2012 to retrieve any potential randomized controlled trials designed to evaluate the clinical effectiveness of ZGXFD for EH reported in any language, with main outcome measure as blood pressure (BP). *Results*. Six randomized trials were included. Methodological quality of the trials was evaluated as generally low. Four trials compared prescriptions based on ZGXFD with antihypertensive drugs. Meta-analysis showed that ZGXFD was more effective in BP control and TCM syndrome and symptom differentiation (TCM-SSD) scores than antihypertensive drugs. Two trials compared the combination of modified ZGXFD plus antihypertensive drugs with antihypertensive drugs. Meta-analysis showed that there is significant beneficial effect on TCM-SSD scores. However, no significant effect on BP was found. The safety of ZGXFD is still uncertain. *Conclusions*. ZGXFD appears to be effective in improving blood pressure and hypertension-related symptoms for EH. However, the evidence remains weak due to poor methodological quality of the included studies. More rigorous trials are warranted to support their clinical use.

## 1. Introduction

Cardiovascular disease (CVD) is the leading cause of mortality all over the world. Despite advances in prevention and treatment over the past 20 years, CVD remains a leading cause of death and disability. The emergence of CVD as a leading cause of morbidity and mortality in China, in large part, is a result of the rapid economic growth and associated sociodemographic change that has occurred over the past few decades [1]. Hypertension is the most common CVD in the world, with a prevalence above 20 percent in the general population [2]. It is the most powerful predictor of stroke, myocardial infarction, heart failure, and renal failure. Prior clinical trials have consistently shown that reductions in blood pressure reduce the incidence of stroke and myocardial infarction [3].

Complementary and alternative medicine (CAM) refers to a series of medical and health care practices and products that are not an integral part of conventional medicine due to insufficient proof of their safety and effectiveness [4, 5]. The number of patients who utilize CAM as a treatment of CVDs continues to grow [6, 7]. Traditional Chinese medicine (TCM) is one of the most important parts in CAM [8]. Many studies have shown that TCM, either herbal medicine or acupuncture, could contribute to blood pressure control [9–12]. Eugene Braunwald, a world leader in cardiology for more than half a century, pointed out that current cardiology practice is evidence-based and global in scope [13]. Thus, it is important to investigate the beneficial and harmful effects of Chinese herbs and formulas in the treatment of hypertension under the guidance of scientific assessment methods. 

Zhen Gan Xi Feng Decoction (ZGXFD), a traditional Chinese herbal formula containing twelve commonly used herbs (achyranthes root, ruddle, dragon bone, oyster shell, plastrum testudinis, white peony root, radix scrophulariae, radix asparagi, fructus toosendan, raw malt, artemisia capillaris Thunb, and glycyrrhiza), is widely used to treat hypertension-related symptoms in clinical practice for centuries in China. Recent researches showed that ZGXFD could contribute to blood pressure control. The mechanism of the prescription maybe related to calming liver, suppressing liver yang hyperactivity, and nourishing kidney yin in Chinese medicine. Biochemically, ZGXFD also showed good effect in decreasing the concentrations of angiotensin in plasma and myocardium, reducing the endothelin content in brain and improving PPAR*γ* mRNA expression in rats with essential hypertension [20, 21]. 

Currently, ZGXFD used alone or combined with antihypertensive drugs has been widely used as an alternative and effective method for essential hypertension treatment in China. And until now a number of clinical studies of ZGXFD reported the effectiveness ranging from case reports and case series to controlled observational studies and randomized clinical trials. However, there is no critically appraised evidence such as systematic reviews or meta-analyses on potential benefit and safety of ZGXFD for essential hypertension to justify their clinical use and their recommendation. Understanding the effect of ZGXFD on blood pressure, quality of life (QOL) and cardiovascular risk factors could be valuable for the management of essential hypertension. The present paper aims to evaluate the beneficial and harmful effects of ZGXFD for treatment of essential hypertension in randomized trials. 

## 2. Methods

### 2.1. Database and Search Strategies

The literature searches were conducted in the Cochrane Central Register of Controlled Trials (CENTRAL) in the Cochrane Library (August, 2012), PubMed, Chinese Biomedical Literature Database (CBM), Chinese National Knowledge Infrastructure (CNKI), Chinese Scientific Journal Database (VIP), and searched the reference list of retrieved papers. All of those searches ended on August 15, 2012. Ongoing registered clinical trials were searched in the website of Chinese clinical trial registry (http://www.chictr.org/) and international clinical trial registry by US National Institutes of Health (http://clinicaltrials.gov/). The following search terms were used individually or combined: “essential hypertension,” “hypertension,” “Zhen Gan Xi Feng Decoction,” “clinical trial,” and “randomized controlled trial.” The bibliographies of included studies were searched for additional references. 

### 2.2. Inclusion Criteria

All the randomized controlled trials (RCTs) of all the prescriptions based on “Zhen Gan Xi Feng Decoction” compared with antihypertensive drugs in patients with hypertension were included. RCTs combined ZGXFD with antihypertensive drugs compared with antihypertensive drugs, and all the modified ZGXFD were included as well. There were no restrictions on population characteristics, language, and publication type.

The primary outcome measure was blood pressure (BP), and the secondary outcome measure was TCM syndrome and symptom differentiation (TCM-SSD) scores. The criteria “significant effective, effective, or not effective” were also included in the outcome measurement. Duplicated publications reporting the same groups of participants were excluded.

### 2.3. Data Extraction and Quality Assessment

Two authors conducted the literature searching (X. J. Xiong and X. C. Yang), study selection (X. J. Xiong and W. Liu), and data extraction (X. J. Xiong and X. Du) independently. The extracted data included authors and title of study, year of publication, study size, age and sex of the participants, details of methodological information, name and component of Chinese herbs, treatment process, details of the control interventions, outcomes (e.g., blood pressure), and adverse effects for each study. Disagreement was resolved by discussion and reached consensus through a third party (J. Wang).

Methodological quality of trials was assessed using 7 criteria from the Cochrane Handbook for Systematic Review of Interventions, Version 5.1.0 (X. J. Xiong and B. Feng) [22]. The items included random sequence generation (selection bias), allocation concealment (selection bias), blinding of participants and personnel (performance bias), blinding of outcome assessment (detection bias), incomplete outcome data (attrition bias), selective reporting (reporting bias), and other bias. The quality of all trials was categorized to low/unclear/high risk of bias (“Yes” for a low of bias, “No” for a high risk of bias, or “Unclear” otherwise). Three levels were used to evaluate the trials: low risk of bias (all the items were in low risk of bias), high risk of bias (at least one item was in high risk of bias), and unclear risk of bias (at least one item was in unclear).

### 2.4. Data Synthesis

RevMan 5.1 software provided by Cochrane Collaboration was used for data analyses. Dichotomous data were expressed as relative risk (RR) and continuous outcomes as weighted mean difference (WMD), both with 95% confidence intervals (CI). Meta-analysis was performed if the intervention, control, and outcome were the same or similar. The statistical heterogeneity was presented as significant when *I* square (*I* ^2^) is over 50% or *P* < 0.1. In the absence of significant heterogeneity, we pooled data using a fixed-effect model (*I* ^2^ < 50%), and otherwise we used random effects model (*I* ^2^ > 50%) [22].

## 3. Result

### 3.1. Description of Included Trials

**Figure 1:**
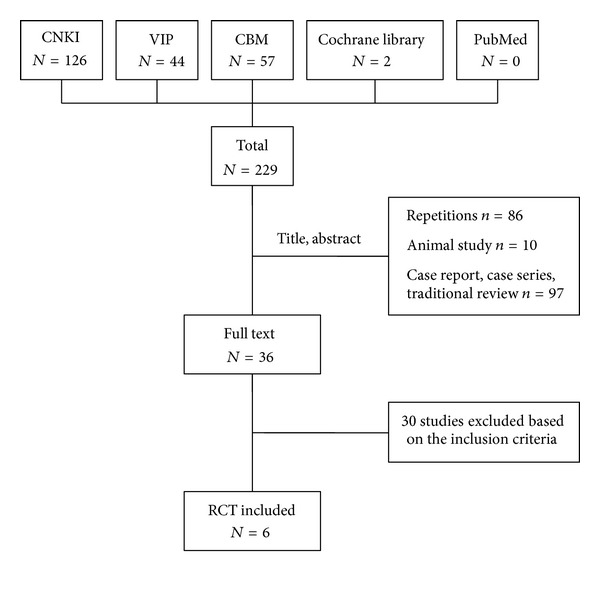
Study selection process.

After primary search of 5 databases, 229 trials were screened out from electronic and manual searches (Figure 1), and the majority were excluded due to obvious ineligibility which included irrelevant titles and abstract (some papers being found from more than one database). After reading the titles and abstracts, a majority of them was excluded. Eighty-six trials were excluded because of duplicated publication, 10 trials were excluded due to the animal studies, and the rest 97 trials were noncontrolled clinical trials including case report, case series traditional review. Thirty out of the rest of 36 articles were excluded based on the inclusion criteria. In the end, 6 RCTs were reviewed [14–19]. All the RCTs were conducted in China and published in Chinese. The characteristics of 6 randomized trials are summarized in Table 1. 

The 6 RCTs involved 830 patients with essential hypertension. There was a wide variation in the age of subjects (18–87 years). Six (6) trials specified three diagnostic criteria of hypertension, one trial [15] used 1999 WHO-ISH guidelines for the management of hypertension (1999 WHO-ISH GMH), one trial [16] used Chinese Guidelines for the Management of Hypertension-1999 (CGMH-1999), one trial [17] used China Guidelines on Prevention and Management of High Blood Pressure-2004 (CGPMHBP-2004), and three trials [14, 18, 19] only demonstrated patients with essential hypertension. Six (6) trials have reported TCM diagnostic criteria with yin-deficiency and excessive yang syndrome, four trials [14–17] used Guidelines of Clinical Research of New Drugs of Traditional Chinese Medicine (GCRNDTCM), and two trials [18, 19] only demonstrated patients with yin-deficiency and excessive yang syndrome in TCM. 

The interventions included all the prescriptions based on “Zhen Gan Xi Feng Decoction” alone and ZGXFD with antihypertensive drugs. The controls included antihypertensive drugs alone. Four trials [14–16, 18] investigated the prescriptions based on “Zhen Gan Xi Feng Decoction” using alone versus antihypertensive drugs, and the remainig two trials [17, 19] compared the prescriptions based on “Zhen Gan Xi Feng Decoction” plus antihypertensive drugs versus antihypertensive drugs.

The total treatment duration ranged from 2 weeks to 4 weeks. The variable prescriptions are presented in Table 1. The different compositions of formula ZGXFD are presented in Table 2. All of the 6 trials used the blood pressure (BP) as the main outcome measure. Other outcome measures include the scale for TCM syndrome and symptom differentiation (TCM-SSD). Adverse effect was described in details. Three classes were used to evaluate treatment effects, including significant effective, effective, and ineffective according to BP and TCM-SSD.

### 3.2. Methodological Quality of Included Trials

The methodological quality of the included trials was assessed to be generally low according to the predefined quality assessment criteria in Table 3. The randomized allocation of participants was mentioned in all the included trials [14–19], and only 2 trials [14, 15] stated the methods for sequence generation including random number table. The other 4 trials [16–19] have not reported the randomized allocation of participants with detailed information. However, insufficient information was provided to judge whether it was conducted properly or not. Allocation concealment and double-blind were not mentioned in all trials. None of trials reported dropout or withdraw. None of trials had a pretrial estimation of sample size, which indicated the lack of statistical power to ensure appropriate estimation of the therapeutic effect. Selective reporting was generally unclear in the RCTs due to the inaccessibility to the trial protocol. All the trials did not mention followup. We contacted the authors for further information but regrettably no information could be gotten.

### 3.3. Effect of the Interventions

#### 3.3.1. “Zhen Gan Xi Feng Decoction” versus Antihypertensive Drugs (Western Medicine)

 Four trials [14–16, 18] compared prescriptions based on “Zhen Gan Xi Feng Decoction” using alone with antihypertensive drugs. 

*Blood Pressure.* Four trials [14–16, 18] used blood pressure decrease to measure the outcome: significant effective (diastolic blood pressure decreased by 10 mmHg reaching the normal range, or diastolic blood pressure has not yet returned to normal but has been reduced ≥ 20 mmHg), effective (diastolic blood pressure decreased to less than 10 mmHg reaching the normal range, or diastolic blood pressure decreased by 10–19 mmHg but did not reach the normal range, or systolic blood pressure decreased ≥ 30 mmHg), and ineffective (not to meet the previous standards). The trial showed significant difference between treatment and control group on the three criteria outcome measurement (RR: 1.93 [1.14, 3.25]; *P* = 0.01). Two trials [14, 15] compared the effectiveness using the blood pressure value, and significant difference was found between treatment and control group in systolic blood pressure (WMD: −7.05 [−10.74, −3.35]; *P* = 0.0002) and diastolic blood pressure (WMD: −6.24 [−8.42, −4.07]; *P* < 0.00001) (Tables 4, 5, and 6). 

*TCM-SSD Scores. *Three trials [14–16] used the TCM-SSD scores to measure the outcome: significant effective (the main symptoms such as headache, dizziness, palpitations, insomnia, tinnitus, and irritability disappear, or TCM-SSD scores reduced rate ≥70%), effective (the main symptoms relieved, or 70% > TCM-SSD scores reduced rate ≥30%), and ineffective (The main symptoms do not change, or TCM-SSD scores reduced rate <30%). Significant difference was found between treatment and control group after treatment. Meta-analysis of three trials showed significant difference in favor of modified ZGXFD compare to antihypertensive drugs (RR: 3.78 [1.82, 7.85]; *P* = 0.0004) (Table 7).

#### 3.3.2. “Zhen Gan Xi Feng Decoction” Plus Antihypertensive Drugs versus Antihypertensive Drugs

Two trials [17, 19] compared the combination of modified ZGXFD plus antihypertensive drugs with antihypertensive drugs. 

*Blood Pressure*. Meta-analysis of two trials [17, 19] showed no significant difference on blood pressure (RR: 1.03 [0.47, 2.25]; *P* = 0.93) (Table 4).

*TCM-SSD Scores. *There is only one trial [17] who reported the TCM-SSD scores decrease. The meta-analysis showed that there is significant beneficial effect on the combination group compare to the antihypertensive drugs using alone (RR: 3.87 [1.18, 12.68]; *P* = 0.03) (Table 7). We cannot obtain more details of the TCM-SSD scores. So, we cannot get the analysis of comparison between groups.

### 3.4. Sensitivity Analysis, Subgroup Analysis, and Publication Bias

 The number of trials was too small to conduct any sufficient additional analysis of sensitivity, subgroup, and publication bias.

### 3.5. Adverse Effect

Three out of six trials mentioned the adverse effect [14, 18, 19]. Three trials reported nine specific symptoms including headache, dry cough, diarrhea, palpitations, neutropenia, nausea, dizziness, sleepiness, and itchy skin. One trial reported adverse effect in captopril group including headache and dry cough [14]. One trial mentioned adverse effect both groups, with diarrhea in modified ZGXFD group and dry cough, palpitations, and neutropenia in captopril group [18]. One trial mentioned adverse effect both groups, with gastrointestinal discomfort, dizziness, sleepiness, and itchy skin in modified ZGXFD plus nitrendipine group and nausea, dizziness, and itchy skin in nitrendipine group [19]. 

## 4. Discussion

Currently, more and more systematic reviews (SRs) and meta-analysis have been conducted to assess the efficiency of Chinese herbal medicine for essential hypertension [23–31]. It is demonstrated that Chinese herbal medicine could not only contribute to low BP smoothly, recover the circadian rhythm of BP, but also improve symptoms and signs especially [32–36]. As an adjunctive treatment to antihypertensive drugs, ZGXFD is a popular TCM formula for the treatment of essential hypertension. And until now, more and more RCTs have been published in Chinese language but have not been evaluated according to the Preferred Reporting Items for Systematic Reviews and Meta-Analyses (PRISMA) standard [37]. This study aims to assess the current clinical evidence of ZGXFD for essential hypertension. Our systematic review suggested that ZGXFD may be effective on blood pressure or improvement of TCM-SSD scores (symptoms and signs). However, according to potential publication bias and low-quality trials, available data are not adequate to draw a definite conclusion of ZGXFD in treating essential hypertension. More specifically, the positive findings should be interpreted conservatively due to the following facts. 

Firstly, all the six trials included in this paper had risk of bias in terms of design, reporting, and methodology. Only two randomized controlled trials (RCTs) [14, 15] stated randomization procedure with table of random number. However, limited information was provided to judge whether randomization was conducted properly and really. For the other 4 trials [16–19], they just mentioned that “patients were randomized into two groups” with no detailed information. All RCTs did not mention allocation concealment. Therefore, we could not exclude the possibility that some of the claimed RCTs are not real RCTs. What is more, these trials, including Mao 2005, Luo 2008, Zhou 2000, Liu and Zhong 2008, and Li and Zheng 2012 [15–19], only have one author or two authors. It is impossible for an RCT to be done properly in terms of randomization procedure and the allocation concealment. It is noteworthy that all the trials did not describe the blinding in details. It directly led to performance bias and detection bias due to patients and researchers being aware of the therapeutic interventions for the subjective outcome measures. All the included RCTs did not report presample size estimation and were not multicenter, large scale RCTs. Therefore, it directly prohibited us to perform meaningful analysis between groups. It is well known that, if poorly designed, all the trials would show larger differences between experimental and control groups than those conducted rigorously [38]. 

Second, there was lack of knowledge for the final indicator at endpoint. As we know, the primary goal of essential hypertension treatment is to reduce mortality or prevent progression to severe complications. However, all the included trials only reported the outcomes such as blood pressure and symptom improvement. None of the trials reported the mortality rate or the incidence of complications. Future RCTs of ZGXFD with appropriate design need to be carried out to measure the mortality and morbidity of hypertension. 

Third, our review found inadequate reporting on adverse events in the included trials. Only two of the six trials reported the adverse effect of ZGXFD or modified ZGXFD briefly, providing limited information. One trial [18] mentioned diarrhea, and the other [19] mentioned gastrointestinal discomfort, dizziness, sleepiness, and itchy skin. The remaining four trials did not mention whether they had monitored adverse effects at all. Therefore, conclusions about the safety of ZGXFD cannot be made from this review due to the limited, inadequate recording and reporting of adverse events. There is a widely accepted perception that it is safe to use herbal medicines for various diseases in China. However, with the increasing reports of liver toxicity and other adverse events associated with Chinese herbal medicines [39–44], the safety of ZGXFD needs to be monitored rigorously and reported appropriately in the future clinical trials.

Fourth, publication and other biases may play an important role in the review. Only trials published in China could be identified and included after conducting comprehensive searches. We tried to avoid language bias and location bias; however, potential publication bias could not be excluded totally. Almost all the RCTs claimed positive effect of ZGXFD though some of them turned out to be negative when analyzed by standard statistical techniques using risk ratios or mean differences. We have conducted extensive searches for unpublished material, but no unpublished “negative” studies were found. 

In summary, the reported effectiveness and safety of ZGXFD for essential hypertension cannot be taken as confirmative conclusion. Due to poorly designed and low-quality methodology, the evidence is still inconclusive. We hope that further RCTs with better research methods as a good approach to evaluate the effectiveness will be needed in ZGXFD for essential hypertension clinical study. 

## Figures and Tables

**Table 1:** Characteristics and methodological quality of included studies.

Study ID	Sample	Diagnosis standard	Intervention	Control	Course (week)	Outcome measure
Guo et al. 2002 [14]	129	Hypertension diagnostic criteria (unclear); GCRNDTCM	ZGXFD	Captopril	4	BP; TCM-SSD; side effect
Mao 2005 [15]	70	1999 WHO -ISH GMH; GCRNDTCM	Modified ZGXFD	Indapamide	3	BP; TCM-SSD
Luo 2008 [16]	45	CGMH-1999; GCRNDTCM	ZGXFD	Captopril	2	BP; TCM-SSD
Liu and Zhong 2008 [17]	120	CGPMHBP-2004; GCRNDTCM	Modified ZGXFD plus benazepril	Benazepril	3	BP; TCM-SSD
Li and Zheng 2012 [18]	166	Hypertension diagnostic criteria (unclear); TCM diagnostic criteria (unclear)	Modified ZGXFD	Captopril	4	BP; side effect
Zhou 2000 [19]	300	Hypertension diagnostic criteria (unclear); TCM diagnostic criteria (unclear)	Modified ZGXFD plus nitrendipine	Nitrendipine	4	BP; side effect

**Table 2:** Composition of formula.

Study ID	Formula	Composition of formula
Guo et al. 2002 [14]	ZGXFD	Achyranthes root, ruddle, dragon bone, oyster shell, plastrum testudinis, white peony root, radix scrophulariae, radix asparagi, fructus toosendan, raw malt, artemisia capillaris thunb, and glycyrrhiza.
Mao 2005 [15]	Modified ZGXFD	Achyranthes root 30 g, ruddle 30 g, uncaria 30 g (put in later), dragon bone 15 g, oyster shell 15 g, plastrum testudinis 15 g, white peony root 12 g, radix scrophulariae 12 g, radix asparagi 12 g, fructus toosendan 9 g, raw malt 20 g, artemisia capillaris thumb 9 g, and glycyrrhiza 6 g.
Luo 2008 [16]	ZGXFD	White peony root 30 g, radix asparagi 15 g, achyranthes root 30 g, ruddle 30 g, dragon bone 30 g, oyster shell 30 g, plastrum testudinis 25 g, radix scrophulariae 15 g, fructus toosendan 10 g, raw malt 10 g, artemisia capillaris thumb 15 g, and glycyrrhiza 10 g
Liu and Zhong 2008 [17]	Modified ZGXFD plus benazapril	White peony root 15 g, radix asparagi 15 g, plastrum testudinis 15 g, oyster shell 15 g, fructus toosendan 6 g, ruddle 30 g, achyranthes root 30 g, radix scrophulariae 15 g, dragon bone 15 g, artemisia capillaris thumb 6 g, raw malt 6 g, and glycyrrhiza 5 g. Severe headache plus chrysanthemum 10 g; insomnia plus pearl shell 15 g and caulis polygoni multiflori 15 g; vexation plus gardenia 10 g and scutellaria baicalensis georgi 10 g; severe phlegm-heat plus pinellia pedatisecta schott 6 g and fritillaria cirrhosa 10 g.
Li and Zheng 2012 [18]	Modified ZGXFD	Radix scrophulariae 15 g, ruddle 30 g, white peony root 15 g, achyranthes root 30 g, radix asparagi 15 g, dragon bone 15 g, oyster shell 15 g, raw malt 6 g, artemisia capillaris thumb 6 g, plastrum testudinis 15 g, fructus toosendan 6 g, and glycyrrhiza 3 g. Vexation plus plaster stone; abundant sputum plus pinellia pedatisecta schott and bamboo bark; slow-weak pulse plus prepared radix rehmanniae and pulp of cornus; diarrhea remove plastrum testudinis and ruddle, plus halloysitum rubrum; insomnia plus coptis chinensis, rehmanniae radix and caulis polygoni multiflori; severe headache plus abalone shell; severe dizziness plus gastrodia elata.
Zhou 2000 [19]	Modified ZGXFD plus nitrendipine	White peony root 20 g, radix asparagi 10 g, plastrum testudinis 5 g, oyster shell 30 g, abalone shell 20 g, ruddle 80 g, magnetite 30 g, achyranthes root 10 g, radix scrophulariae 15 g, salvia miltiorrhira 30 g, and panpax notoginseng 10 g. Severe headache plus antelope horn; insomnia plus pearl shell and caulis polygoni multiflori; vexation plus gardenia and scutellaria baicalensis georgi; severe phlegm-heat plus pinellia pedatisecta schott and fritillaria cirrhosa.

**Table 3:** Quality assessment of included randomized controlled trials.

Included trials	Sequence generation	Allocation concealment	Blinding of participants personnel and outcome assessors	Incomplete outcome data	Selective outcome reporting	Other sources of bias	Risk of bias
Guo et al. 2002 [14]	Table of random number	Unclear	Unclear	No	No	Unclear	Unclear
Mao 2005 [15]	Table of random number	Unclear	Unclear	Yes	No	Unclear	Unclear
Luo 2008 [16]	Unclear	Unclear	Unclear	Yes	No	Unclear	High
Liu and Zhong 2008 [17]	Unclear	Unclear	Unclear	Yes	No	Unclear	High
Li and Zheng 2012 [18]	Unclear	Unclear	Unclear	Yes	No	Unclear	High
Zhou 2000 [19]	Unclear	Unclear	Unclear	Yes	No	Unclear	High

**Table 4:** Analyses of blood pressure.

Trials		Intervention (*n*/*N*)	Control (*n*/*N*)	RR [95% CI]	*P* value
ZGXFD versus antihypertensive drugs					
ZGXFD versus captopril	1	54/68	40/61	2.02 [0.92, 4.46]	0.08
Modified ZGXFD versus indapamide	1	40/47	19/23	1.20 [0.31, 4.61]	0.79
ZGXFD versus captopril	1	22/24	18/21	1.83 [0.28, 12.19]	0.53
Modified ZGXFD versus captopril	1	78/86	65/80	2.25 [0.90, 5.64]	0.08

Meta-analysis	4	194/225	142/185	1.93 [1.14, 3.25]	0.01

ZGXFD plus antihypertensive drugs versus antihypertensive drugs					
Modified ZGXFD plus benazapril versus benazapril	1	56/60	47/60	3.87 [1.18, 12.68]	0.03
Modified ZGXFD plus nitrendipine versus nitrendipine	1	183/200	72/100	0.07 [0.00, 1.22]	0.07

Meta-analysis	2	239/260	119/132	1.03 [0.47, 2.25]	0.93

**Table 5:** Analyses of systolic blood pressure.

Trials		MD [95% CI]	*P* value
ZGXFD versus antihypertensive drugs			
ZGXFD versus captopril	1	−10.94 [−15.64, −6.24]	<0.00001
Modified ZGXFD versus indapamide	1	−0.74 [−6.72, 5.24]	0.81

Meta-analysis	2	−7.05 [−10.74, −3.35]	0.0002

**Table 6:** Analyses of diastolic blood pressure.

Trials		MD [95% CI]	*P* value
ZGXFD versus antihypertensive drugs			
ZGXFD versus captopril	1	−8.42 [−10.98, −5.86]	<0.00001
Modified ZGXFD versus indapamide	1	−0.52 [−4.67, 3.63]	0.81

Meta-analysis	2	−6.24 [−8.42, −4.07]	<0.00001

**Table 7:** Analyses of TCM-SSD Scores.

Trials		Intervention (*n*/*N*)	Control (*n*/*N*)	RR [95% CI]	*P* value
ZGXFD versus antihypertensive drugs					
ZGXFD versus captopril	1	59/68	44/61	2.53 [1.03, 6.21]	0.04
Modified ZGXFD versus indapamide	1	45/47	17/23	7.94 [1.46, 43.24]	0.02
ZGXFD versus captopril	1	23/24	15/21	9.20 [1.00, 84.26]	0.05

Meta-analysis	3	127/139	76/105	3.78 [1.82, 7.85]	0.0004

BBTD plus antihypertensive drugs versus antihypertensive drugs					
modified ZGXFD plus benazapril versus benazapril	1	56/60	47/60	3.87 [1.18, 12.68]	0.03

Meta-analysis	1	56/60	47/60	3.87 [1.18, 12.68]	0.03
